# Effects of Peroxiredoxin 6 and Its Mutants on the Isoproterenol Induced Myocardial Injury in H9C2 Cells and Rats

**DOI:** 10.1155/2022/2576310

**Published:** 2022-03-26

**Authors:** Runhong Mu, Siping Ye, Rui Lin, Yupeng Li, Xiao Guo, Liping An

**Affiliations:** ^1^Basic Medical College of Beihua University, Jilin, Jilin 132013, China; ^2^Pharmacy College of Beihua University, Jilin, Jilin 132013, China

## Abstract

**Background:**

Peroxiredoxin 6 (PRDX6) is an important antioxidant enzyme, with a potential application value in the treatment of diseases caused by oxidative damage.

**Methods:**

PRDX6 and a mutant (mPRDX6) were heterologously expressed by using an E.coli expression system and purified by Ni-affinity chromatography. Isoproterenol (ISO) was used to induce a myocardial cell injury model and an animal myocardial injury model. After the treatment with PRDX6 and mPRDX6, the proliferation activity of H9C2 cells was detected by Cell Counting Kit-8 (CCK8) method; the apoptosis was evaluated by flow cytometry, and the histological changes of myocardial cells were observed by hematoxylin and eosin (H&E) staining, the levels of catalase (CAT), glutathione peroxidase (GPX), malondialdehyde (MDA), and superoxide dismutase (SOD) in ISO-treated H9C2 cells as well as in the heart tissue and serum of rats treated with ISO were detected, and the expression levels of Bax, Bcl-2 and peroxisome proliferators-activated receptors-*γ* (PPAR-*γ*) proteins were detected by Western blot.

**Results:**

PRDX6 and mPRDX6 were successfully expressed and purified. The results of efficacy study showed that the mutant mPRDX6, in which the phospholipaseA2 (PLA2) activity of PRDX6 was deleted by site directed mutation, had a better protective effect against the myocardial injury than PRDX6. CCK8 results showed that compared with that in ISO group, the proliferation activity of H9C2 cells was significantly enhanced (*P* < 0.01), the apoptosis rate was significantly decreased (*P* < 0.01), and the fluorescence intensity of reactive oxygen species (ROS) was significantly decreased (*P* < 0.01) in mPRDX6 group. The results of H&E staining showed that the myocardial injury was alleviated to a certain extent in mPRDX6 group. Compared with those in ISO group, the activities of CAT, GPX, and SOD in H9C2 cells and the heart tissue and serum of rats were significantly increased (*P* < 0.05), while the contents of MDA were significantly decreased (*P* < 0.05). Western blot analysis showed that the expression level of Bcl-2 in H9C2 cells was significantly decreased (*P* < 0.01), and that of Bax and PPAR-*γ* was significantly increased (*P* < 0.05).

**Conclusion:**

mPRDX6 has a protective effect against the myocardial injury induced by ISO, and the mechanism may be related to its antioxidation. This study may provide a theoretical basis for the research and development of drugs used for the treatment of myocardial injury.

## 1. Introduction

Reactive oxygen species (ROS), as a key signal transduction molecule in many important metabolic and regulatory pathways, is involved in the development, growth, differentiation, and proliferation of cells. However, when ROS is produced too much and cannot be scavenged in time, it will produce irreversible oxidative modification on DNA, proteins, and lipids, and then can cause the mutation of genes, leading to a series of diseases [1, 2]. There is a complete set of antioxidant defense enzyme system in the human body to prevent the harmful effects of ROS on the human body. The synergy between them can not only effectively scavenge the excessive ROS to maintain it in the normal level but also timely repair and clean up the biological molecules damaged by oxidation to effectively protect cells from oxidative damage. However, with the increase of environmental pollution, electromagnetic radiation, enormous social pressure, and drug abuse, ROS is excessive and cannot be scavenged in time, leading to many diseases [3]. Ischemic heart disease is a cardiovascular disease which seriously endangers human health, and the incidence of ischemic heart disease is increasing year by year with the change of people's lifestyle and diet. The theory of oxygen free radicals is one of the main mechanisms for ischemic heart disease [4, 5]. It is believed based on this theory that in myocardial ischemia, the blood perfusion of the heart decreases and then cause an insufficient oxygen supply to the heart, so the myocardial aerobic metabolism cannot proceed normally, in which the ability of myocardial tissue to generate ROS far exceeds the body's ability to scavenge it, resulting in a rapid excessive accumulation of ROS. Peroxiredoxin (PRDX), an important family of antihydrogen peroxide proteins widely existing in organisms, can effectively protect cells from peroxidation damage, regulate signal transduction pathways, and inhibit the growth of tumor cells based on its antioxidant properties [6]. The study of spinal cord injury model indicated that an increased expression of PRDX1 could inhibit the activity of ROS, mitigate the injury of cells, and promote their functional recovery [7]. It was found in the study of myocardial infarction model that the overexpression of PRDX2 could induce a decreased production of ROS, alleviating the myocardial injury caused by myocardial infarction [8]. Many studies have shown that PRDX family is involved in biological processes such as oxidative stress, cell damage, and myocardial ischemia [9]. PRDX6 is the only1-Cys member with gonad-stimulating hormone (GSH) as electron donor in PRDX family, with dual enzyme activities of both glutathione peroxidase (GPX) and phospholipase A2 (PLA2), and its main function in the body is to regulate redox reaction and phospholipid metabolism [10]. Moreover, PRDX6 plays an important protection role in the pathological state of different oxidative injuries of skin, lung, eyes, gastrointestinal tract, nervous system, and ovary [11–13]. However, the role of PRDX6 in myocardial ischemia is still unclear. Isoproterenol (ISO), a*β* receptor agonist, can increase the oxidative stress induced by ROS in cardiomyocytes and eventually cause an oxidative stress injury of the heart. The aim of this study was to investigate the protective effect of PRDX6 against ISO-induced myocardial injury and whether the PLA2 activity of PRDX6 could affect its protective effect. Firstly, PRDX6 gene was extracted to construct a plasmidColdI (pColdI)-PRDX6 expression vector, and on this basis, Ser32 was mutated into Ala [14], so that the PLA2 activity of PRDX6 could be deleted without affecting its GPX activity to construct a mutant (mPRDX6). Then, PRDX6 and mPRDX6 were heterologously expressed by using an E.coli expression system, and the protective effect of PRDX6 and mPRDX6 against the ISO-induced myocardial injury in H9C2 cells and Sprague Dawley (SD) rats was investigated in order to explore their potential application value.

## 2. Experimental Materials and Methods

### 2.1. Experimental Materials

Dimethylol dimethyl (DMDM) medium (GIBCO, USA); fetal bovine serum (GIBCO, USA); isoproterenol (Shanghai Hefeng, China); CCK8 kit (Biyuntian Biotechnology Co., Ltd., China); Annexin V-FITC/7-Amino-Actinomycin D(V-FITC/7-AAD) kit (Sino biological, China); Catalase (CAT) kit, GPX kit, MDA kit, and superoxide dismutase (SOD) kit (Nanjing Jiancheng Bioengineering Institute, China); peroxisome proliferater-activated receptor gamma (PPAR-*γ*) antibody, B-cell lymphama-2 (Bcl-2) antibody and BCL2-associated X (Bax) antibody (Abcam, USA); goat anti-mouse horseradish peroxidase (HRP) (Boster Company, China). *E. coli* DH5 *α* and BL21 (DE3) engineering strain, pColdI vector and human gastric cDNA library were preserved in our laboratory. Animal experiments were approved by IACUC of Beihua University, and all rat experiments were carried out in accordance with Regulations on The Management of Experimental Animals (Revised in 2017) (State Council Order No. 676). Thirty two male SD rats, weighing 170–190 g, were used in the experiments.

### 2.2. Experimental Methods

#### 2.2.1. Construction of PRDX6 Recombinant Plasmid and Mutant (mPRDX6)

Using human gastric cDNA library as the template of polymerase chain reaction (PCR), the primers were designed according to the reported sequence of PRDX6 gene, PRDX6-F: 5′-CCCAAGCTTATGCCCGGAGGTC-3′ and PRDX6-R: 5′-GCTCTAGATTAAGGCTGGGGTG-3′. PCR reaction conditions: denaturation at 94°C for 2 min, denaturation at 94°C for 50 s, annealing at 52–57°C for 45 s and extension at 72°C for 1 min with 35 cycles, kept at 72°C for 8 min, and kept at 4°C. The PCR products, PRDX6 and plasmid pColdI, were digested with H*ind* III/X*ba* I double enzyme, and the digested fragments were recovered using a gel recovery kit. The PRDX6 gene fragment and plasmid were connected at 16°C overnight according to the molar ratio of 5 : 1. The connection product was transformed in E. coli DH5*α* competent cells, and the positive transformant were screened on a Luria-Bertani (LB) solid medium containing ampicillin resistance and identified by double enzyme digestion and PCR.

Using pColdI-PRDX6 as template, Ser32 was mutated into Ala. The mutation primers were S32A PRDX6-F: 5′-CATGGGGCATTCTCTTCTCCCACC-3′ and PRDX6-R: 5′-CATGGGGCATTCGCTTCTCCCACC-3′. The mutation PCR reaction conditions: 95°C for 2 min, 95°C for 30 s, 55°C for 90 s, and 68°C for 12 min with 16 cycles and kept at 4°C. The template plasmid of PCR products was digested with DpnI enzyme at 37°C for 16 h. The positive transformants were screened on the LB solid medium containing ampicillin resistance. Finally, the plasmid extracted from the positive clone was sent to Shanghai Bioengineering Co., Ltd. for sequencing.

#### 2.2.2. Expression and Purification of PRDX6 and mPRDX6 in E. coli

The two recombinant plasmids in Section 2.1 were transformed into BL21 (DE3) competent cells and cultured at 37°C overnight. The positive transformants were screened on the LB solid medium containing ampicillin resistance. The positive clones were selected and cultured in 50 mL medium. When OD600 was 0.6, isopropyl *β*-d-thiogalactoside (IPTG) was added to induce the positive clones for 2 h for the collection of bacteria. The bacteria were resuspended in PBS and the suspension was centrifuged at 4°C and 5000 g for 5 min, and the bacteria were broken by ultrasound in an ice water bath and centrifuged at 4°C for 10 min to collect the protein supernatant. The Ni^2+^ chelating column was prepared and the proteins were purified according to the method provided in the instructions of Chelating Sepharose Fast Flow. The nonspecific adsorbed heteroproteins were eluted, respectively, with 5-fold column volume elution buffer containing 50 mmol/L, 100 mmol/L, and 150 mmol/L imidazole, and then the target proteins were eluted with 5-fold column volume elution buffer containing 300 mmol/L imidazole. The eluent containing the target proteins was dialyzed with a dialysis bag with a molecular weight cutoff of 8000–10000 to remove salt and imidazole. Finally, the target proteins were concentrated with polyethylene glycol 20000. The purified proteins were identified by sodium dodecyl sulfate-polyacrylamide gel electrophoresis (SDS-PAGE) and Western blot.

#### 2.2.3. Treatment and Grouping of H9C2 Cells

H9C2 cells were cultured in DMEM medium supplemented with 10% fetal bovine serum and penicillin-streptomycin (double antibody) (penicillin 100 U·mL^−1^, streptomycin 100 mg·L^−1^) at 37°C, in 5% CO_2_ and with 95% saturated humidity. The next day, the culture medium was changed and the cells were continued to be cultured, and when the cell coverage rate reached 90%, the cells were subcultured. It was found in our previous experiment that 0.1 mg/mL ISO could be used to treat H9C2 cells for 24 h to construct a H9C2 cell injury model, and the optimal concentration of PRDX6 and mPRDX6 was 0.1 *μ*g/mL.

H9C2 cells were divided into four groups: control (CON) group, in which the cells were treated with an equal volume of PBS, ISO group, in which the cells were treated with 0.1 mg/mL ISO for 24 h, PRDX6 group, in which the cells were pretreated with PRDX6 medium for 2 h and then with 0.1 mg/mL ISO for 24 h, and mPRDX6 group, in which the cells were pretreated with a medium containing mPRDX6 for 2 h and then with 0.1 mg/mL ISO for 24 h, followed by the subsequent experiments.

#### 2.2.4. Proliferation Assay of H9C2 Cells

H9c2 cells were seeded into 96-well plates at a density of 3 × 10^5^ cells/mL, and 3 parallel wells were set in each group. The cells were grouped and treated as described in Section 2.2.3. After the culture for 24 h, CCK8 solution was added into the wells and the cells were incubated for 4 h. Then, the supernatant was discarded, and 150 mL DMSO was added into each well and the plates were vibrated for 5 min to dissolve the crystalline substance. The absorbance was measured at 490 nm by a microplate reader.

#### 2.2.5. Detection of ROS in H9C2 Cells

H9C2 cells were treated as described in Section 2.2.3 and cultured for 24 h. Then, the medium was replaced by dichlorodihydrofluorescein-diacetate 10 (DCF-DA10)*μ*mol/L serum-free medium and the cells were incubated at 37°C for 20 min, and then the culture medium was discarded and the cells were observed under a fluorescence microscope. At the same time, the cells were harvested and the cell density was adjusted to 2 × 10^6^/mL, and 100 *μ*L of the cell suspension were added into each well in black 96-well plates and 3 parallel wells were set in each group. A fluorescence microplate was used to measure the *λ* value of each well, and the excitation wavelength was 488 nm and the emission wavelength was 525 nm.

#### 2.2.6. Detection of the Apoptosis Rate by Flow Cytometry

H9C2 cells were grouped into four groups and treated as described in Section 2.2.3, and cultured for 24 h. The cells were collected and washed twice with PBS precooled at 4°C. After centrifugation, the supernatant was discarded and the cells were washed in the same way, and then the cells were suspended with 1 × binding buffer at a cell density of 1 × 10^7^/mL. The cell suspension was transferred into labeled flow cytometry tubes, then 5 *μ*L annexin V-FITC and 5 *μ*L l7-AAD were added into each of the tubes, mixed gently, and the cells were incubated at room temperature in darkness for 15 min. A negative control group (unstained cells) and a single dye staining control group (stained with only one dye) were set for setting the voltage of flow cytometry and its compensation, and then the apoptosis rate of the cells was detected. Finally, the data were analyzed and plotted with FlowJo V10.

#### 2.2.7. Grouping and Administration of Experimental Animals

All rats were raised under the standard feeding conditions. Based on the previous experimental results, the administration dosage of PRDX6 and mPRDX6 was 2 *μ*g/kg·d. The rats were randomly divided into four groups, 8 in each group. An animal model of myocardial ischemia injury was established by the subcutaneous injection of Isoprenaline (ISO). Rats in CON group were subcutaneously injected with an equal volume of normal saline, while those in ISO, PRDX6, and mPRDX6 groups were subcutaneously injected with 80 mg/kg ISO successively for 7 days, and after the modeling, rats in PRDX6 group were subcutaneously injected with 2 *μ*g/kg·d PRDX6 successively for 14 days, and those in mPRDX6 group were injected with 2 *μ*g/kg·d mPRDX6 in the same way.

#### 2.2.8. Detection of Biochemical Indicators

H9C2 cells were grouped and treated as described in Section 2.2.3, then the supernatant was discarded and the cells were digested with trypsin, and the cell solution was centrifuged at 1000 rpm^−1^ for 10 min to collect the cells. The cells were added with 1 mL PBS, then broken and homogenized manually in an ice water bath for 3 min, and the cell homogenate was taken for use. The rats were anesthetized with an excessive dose of barbital (100 mg), and the blood was collected by cardiac puncture. The blood was centrifuged at 3500 rpm^−1^ for 10 min, and the serum was taken for standby. Then, the rats were sacrificed and the heart tissue was taken for the follow-up experiments. CAT, GPX, MDA, and SOD in the H9C2 cells, serum, and heart tissue were detected.

#### 2.2.9. Hematoxylin and Eosin (H&E) Staining

The heart tissue was fixed in a phosphate solution containing 10% formaldehyde and ethylene diamine tetra acetic Acid (EDTA) decalcified buffer at 37°C for the preparation of paraffin sections. The paraffin sections were dewaxed with xylene and dehydrated with gradient ethanol solution, then stained with hematoxylin for 6 min, followed by the differentiation with hydrochloric acid ethanol solution, stained with eosin for 10 min, and finally dehydrated with gradient ethanol solution and then mounted with neutral gum after xylene treatment, in which the sections were stained after drying at room temperature.

#### 2.2.10. Detection of the Expression of Related Proteins by Western Blot

Bicinchoninic acid (BCA) method was used to determine the protein concentration of the samples. 10 *μ*g of protein samples from each group were mixed with 5× protein loading buffer, then boiled at 100°C for 5 min to denature the proteins. After SDS-PAGE, the polyvinylidene fluoride (PVDF) membrane was blocked with TBST solution containing 5% skimmed milk powder at room temperature for 2 h. The PVDF membrane was incubated at 4°C with the primary antibody diluents (1 : 2000) overnight and washed with Tris Buffered Saline with Tween (TBST) 3 times, 5 min each time, and then incubated with the second antibody at 37°C for 1 h and washed with TBST 3 times in the same way. ECL chemiluminescence solution was added on the membrane for its development and photograph. With glyceraldehyde-3-phosphate dehydrogenase (GADPH) protein as internal reference, the optical density of protein bands was measured with ImageJ software, and the final results were expressed as the ratio of target band and internal reference GADPH.

#### 2.2.11. Statistical Analysis

All data were expressed as mean ± standard deviation (mean ± *s*). SPSS 16.0 software was used for the statistical analysis, and differences among groups were tested by means of two-way ANOVA/Bonferroni tests. *P* < 0.05 was considered to be statistically significant.

## 3. Results

### 3.1. Construction of PRDX6 and mPRDX6 Vectors and Their Protein Purification

**Figure 1:**
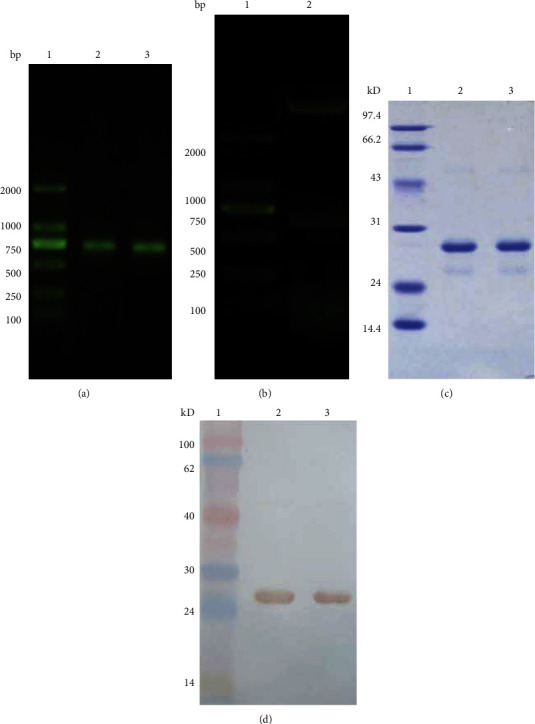
Electrophoresis analysis of PRDX6. (a) Agarose gel electrophoresis analysis of PRDX6. 1: marker DL2000; 2–3: PCR products of PRDX6. (b) Agarose gel electrophoresis analysis of pColdI-PRDX6 digested with H*ind* III/X*ba* I. 1: marker DL2000; 2: pColdI-PRDX6 digested with H*ind* III/X*ba* I.(c) SDS-PAGE analysis of PRDX6 and mPRDX6. (d) Western blot analysis of PRDX6 and mPRDX6.

The agarose gel electrophoresis on the amplified products of PCR was performed. As shown in Figure 1(a), a band between 500–750 bp was consistent with that of known PRDX6 gene fragment with 686 bp in size, without other nonspecific amplification. The results of restriction enzyme digestion of recombinant plasmid shown in Figure 1(b) showed that there were two bands after double restriction enzyme digestion, and the band below them was consistent with the target gene fragment in size. The sequencing was consistent with the target gene sequence, indicating that the recombinant plasmid pCold I-PRDX6 was constructed successfully. The purified PRDX6 and mPRDX6 proteins were detected by SDS-PAGE and Western blot. The SDS-PAGE results showed that there was a band with a molecular weight about 25 KD between 24–31 kD for PRDX6 and mPRDX6, consistent with the molecular weight of their proteins (Figure 1(c)). Western blot further confirmed that PRDX6 and mPRDX6 were successfully expressed in E. coli BL21 (Figure 1(d)).

### 3.2. Effects of PRDX6 and mPRDX6 on the ISO-Induced Injury of H9C2 Cells

**Figure 2:**
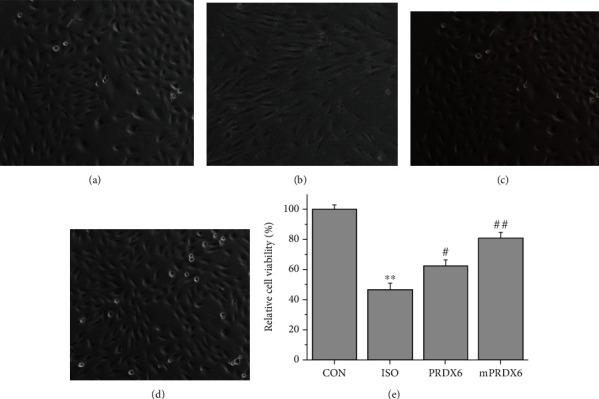
Observation on H9c2 cells under a microscope (200×) (a–d) and relative cell activity (e).(a) CON group; (b) ISO group; (c) PRDX6 group; and (d) mPRDX6 group. ^∗∗^*P* < 0.01 vs. CON group; ^#^*P* < 0.05 vs. ISO group; and ^##^*P* < 0.01 vs. ISO group.

The results of H9C2 myocardial cell injury model test are shown in Figure 2. Compared with that in CON group, the volume of H9C2 cells was reduced, the cell membrane was thickened with wrinkles, and the cytoplasm was tight in ISO group. Compared with those in ISO group, the cells in PRDX6 and mPRDX6 groups recovered normal in morphology to some extent. The CCK8 results showed that the proliferation ability of H9C2 cells in ISO group was significantly lower than that in CON group (*P* < 0.01). Compared with that in ISO group, the cell proliferation activity of H9C2 cells in PRDX6 and mPRDX6 groups was significantly increased (*P* < 0.05), and the cell proliferation activity of mPRDX6 group was higher than that in PRDX6 group (*P* < 0.05) (Figure 2(e)).

### 3.3. Effects of PRDX6 and mPRDX6 on the ISO-Induced Apoptosis of H9C2 Cells

**Figure 3:**
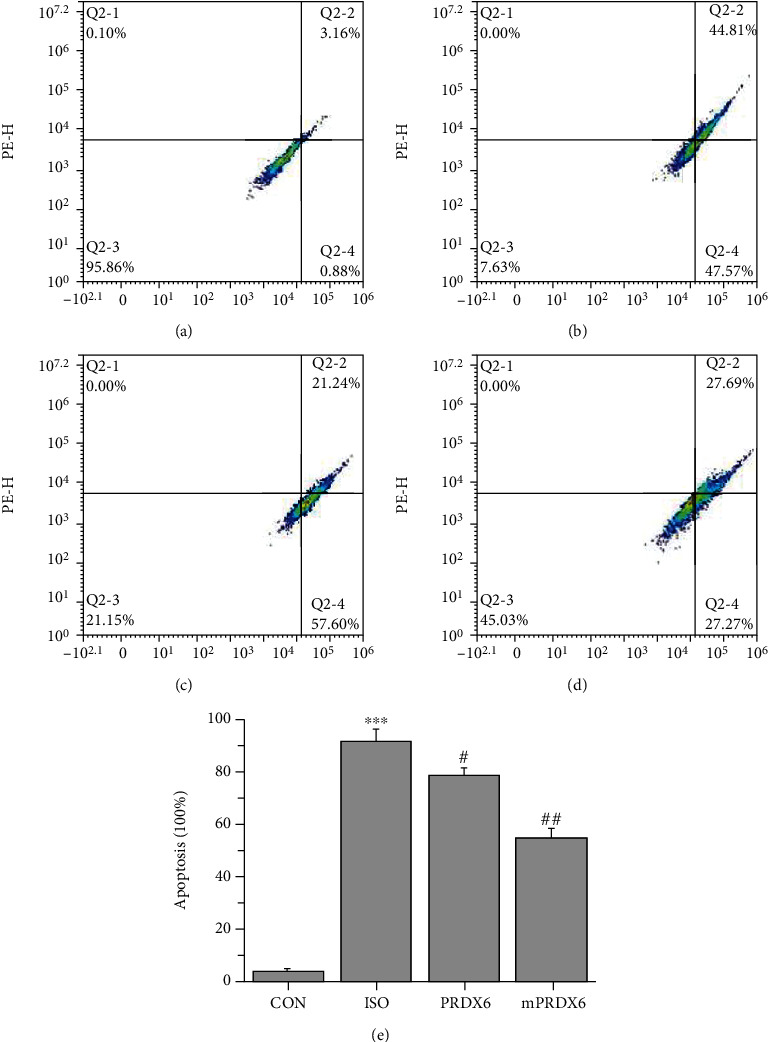
Cell apoptosis results. (a) CON group; (b) ISO group; (c) PRDX6 group; (d) mPRDX6 group; and (e) apoptosis rates in the different groups. ^∗∗∗^*P* < 0.001 vs. CON group; ^#^*P* < 0.05 vs. ISO group; and ^##^*P* < 0.01 vs. ISO group.

Flow cytometry was used to evaluate the effects of PRDX6 and mPRDX6 on the ISO-induced apoptosis of H9C2 cells (Figure 3). Compared with that in CON group, the apoptosis rate of H9C2 cells in ISO group was significantly increased (*P* < 0.001); compared with that in ISO group, the apoptosis rate of H9C2 cells in PRDX6 (*P* < 0.05) and mPRDX6 (*P* < 0.01) groups was significantly decreased, in which compared with that in ISO group, the early apoptosis rate was increased, and the late apoptosis rate was decreased significantly in PRDX6 group, while both the early apoptosis rate and the late apoptosis rate were significantly decreased in mPRDX6 group, indicating that PRDX6 and mPRDX6 could inhibit the apoptosis of H9C2 cells induced by ISO in different ways.

### 3.4. Effects of PRDX6 and mPRDX6 on the ROS in H9C2 Cells Induced by ISO

**Figure 4:**
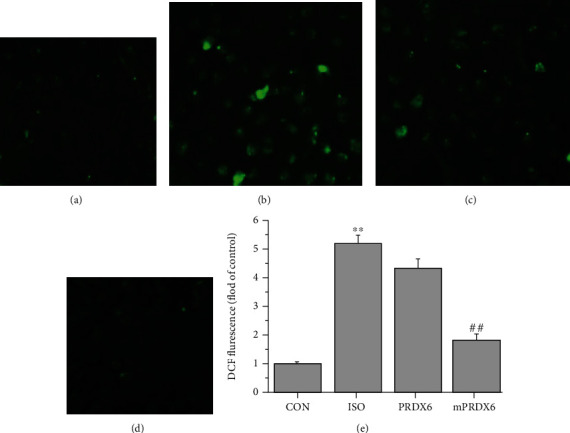
DCFH-DA fluorescence staining results. (a) CON group; (b) ISO group; (c) PRDX6 group; (d) mPRDX6 group; and (e) fluorescence staining intensities in the different groups. ^∗∗^*P* < 0.01 vs. CON and ^##^*P* < 0.01 vs. ISO.

The results of DCFH-DA fluorescence staining are shown in Figure 4. The fluorescence intensity in ISO group, PRDX6 group, and mPRDX6 group was 5.2 times, 4.3 times, and 1.8 times in CON group, respectively. Compared with that in CON group, the fluorescence intensity in ISO group was significantly enhanced (*P* < 0.01) and compared with that in ISO group, and the fluorescence intensity of mPRDX6 group was significantly decreased (*P* < 0.01).

### 3.5. Effects of PRDX6 and mPRDX6 on CAT, GPX, and SOD Enzyme Activities and MDA levels in H9C2 Cells

**Figure 5:**
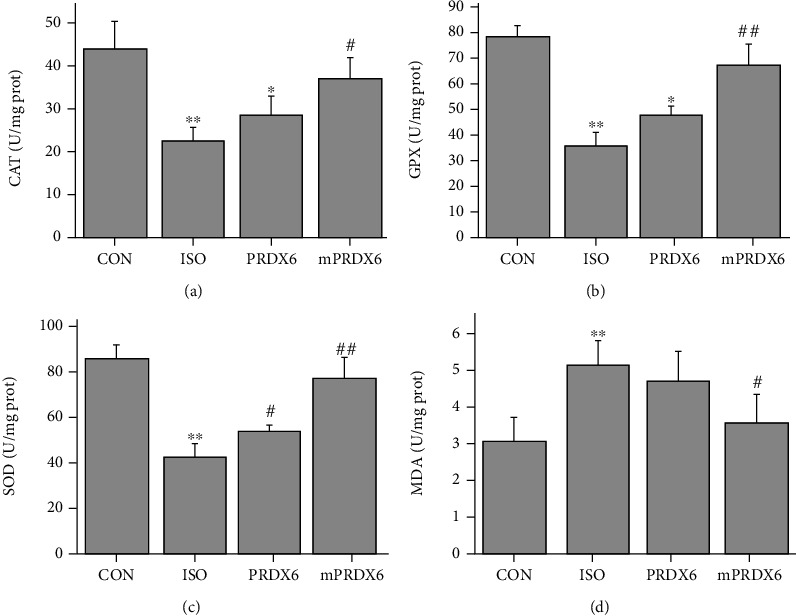
Detection of biochemical indicators in H9C2 cells. (a) CAT activities; (b) GPX activities; (c) SOD activities; and (d) MDA contents. ^∗^*P* < 0.05 vs. CON group; ^∗∗^*P* < 0.01 vs. CON group; ^#^*P* < 0.05 vs. ISO group; and ^##^*P* < 0.01 vs. ISO group.

As shown in Figure 5, compared with those in CON group, the activities of CAT, GPX, and SOD inH9C2 cells were significantly decreased (*P* < 0.01), and the contents of MDA were significantly increased in ISO group (*P* < 0.01); compared with that in ISO group, the activities of CAT, GPX, and SOD in H9C2 cells were significantly increased (*P* < 0.05), and the contents of MDA were significantly decreased in mPRDX6 group (*P* < 0.05); compared with those in ISO group, the activities of CAT and SOD were significantly increased (*P* < 0.05), while the activity of GPX and the content of MDA were not significantly different between ISO group and PRDX6 group (*P* > 0.05).

### 3.6. Effects of PRDX6 and mPRDX6 on the Body Weight and Behavior of Rats Treated with ISO

In contrast to CON group, the activity of rats in ISO group decreased significantly five days after the injection of ISO, occasionally accompanied with some symptoms, such as dyspnea. As shown in Table 1, compared with that in CON group, the body weight of rats in ISO group increased more slowly; and compared with that in ISO group, the activity of rats was significantly recovered, and the body weight was significantly increased in mPRDX6 group (*P* < 0.05).

### 3.7. H&E Staining Results of Rat Heart Tissue

**Figure 6:**
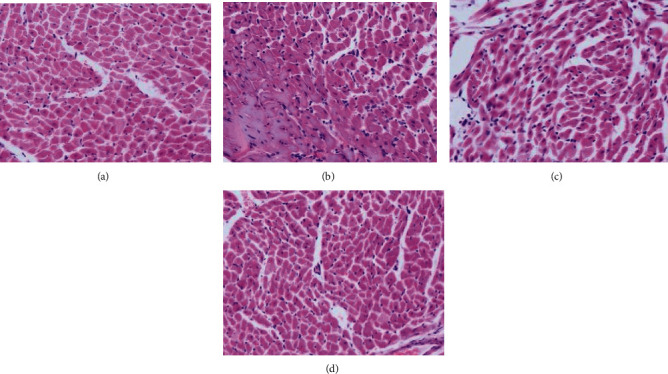
H&E staining of the heart tissue of rats (400×).(a) CON group; (b) ISO group; (c) PRDX6 group; and (d) mPRDX6 group.

H&E staining results (Figure 6) showed that the myocardial cells in CON group were arranged in order and stained evenly, while the myocardial tissue fibers were broken and disordered, and some of the myocardial cells were broken in ISO group; compared with ISO group, the myocardial injury in mPRDX6 group recovered to some extent.

### 3.8. Effects of PRDX6 and mPRDX6 on Serum Biochemical Indicators in Rats

**Figure 7:**
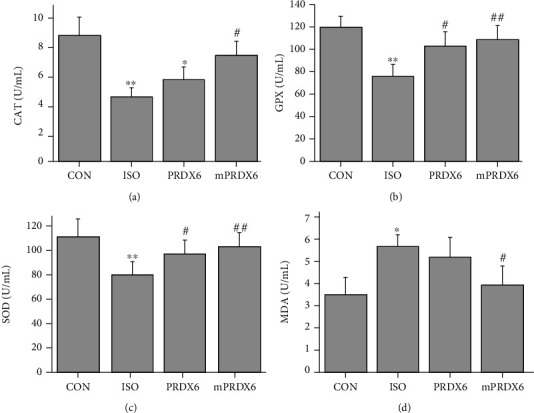
Detection of serum biochemical indicators. A. CAT activities; B. GPX activities; C. SOD activities; D. MDA contents. ∗*P* < 0.05, *vs*.CON group; ∗∗*P* < 0.01, *vs*. CON group; ^#^*P* <0.05, *vs*. ISO group; ^##^*P* <0.01, *vs*. ISO group.

As shown in Figure 7, compared with those in CON group, the activities of SOD, CAT and GPX in the serum of rats in ISO group were significantly decreased (*P* < 0.05); and the contents of MDA were significantly increased (*P* < 0.05); compared with those in ISO group, the activities of CAT, GPX, and SOD in the serum of rats in PRDX6 group and mPRDX6 group were significantly higher (*P* < 0.05), and the contents of MDA in mPRDX6 group were significantly lower (*P* < 0.05), while there was no significant difference in the content of MDA between ISO group and PRDX6 group (*P* >0.05).

### 3.9. Effects of PRDX6 and mPRDX6 on Biochemical Indicators in the Heart Tissue of Rats

**Figure 8:**
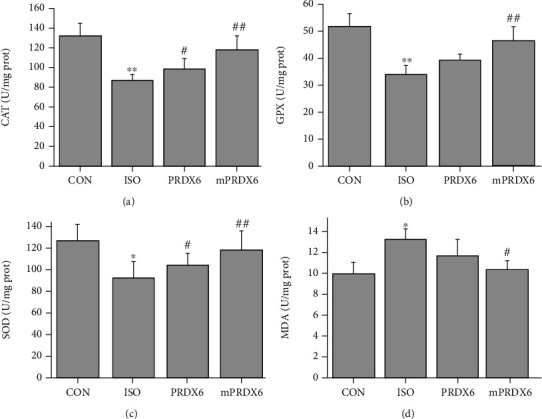
Detection of biochemical indicators in the heart tissue of rats (*n* = 8). (a) CAT activities; (b) GPX activities; (c) SOD activities; and (d) MDA contents. ^∗^*P* < 0.05 vs. CON group; ^∗∗^*P* < 0.01 vs. CON group; ^#^*P* < 0.05 vs. ISO group; and ^##^*P* < 0.01 vs. ISO group.

As shown in Figure 8, compared with those in CON group, the activities of SOD, CAT, and GPX in the heart tissue of rats in ISO group were significantly reduced (*P* < 0.05), and the contents of MDA were significantly increased (*P* < 0.05); compared with those in ISO group, the activities of SOD, CAT, and GPX in the heart tissue of rat in mPRDX6 group were significantly increased (*P* < 0.01), and the contents of MDA were significantly decreased (*P* < 0.05); compared with ISO group, the activities of cat and SOD in heart tissue of rats in PRDX6 group were significantly increased (*P* < 0.05), while there was no significant difference in the activity of GPX and the content of MDA between PRDX6 group and ISO group (*P* > 0.05).

### 3.10. Effects of PRDX6 and mPRDX6 on Expression Levels of Bax, Bcl-2, and PPAR-*γ* Proteins in ISO-Induced H9C2 Cells

**Figure 9:**
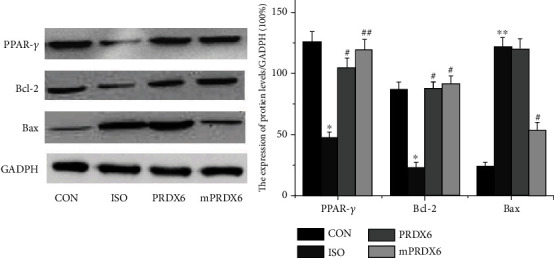
Western blot analysis of expression levels of PPAR-*γ*, Bcl-2, and Bax proteins. ^∗^*P* < 0.05 vs. CON group; ^∗∗^*P* < 0.01 vs. CON group; ^#^*P* < 0.05 vs. ISO group; and ^##^*P* < 0.01 vs. ISO group.

As shown in Figure 9, compared with that in CON group, the expression level of Bcl-2 was significantly decreased and that of Bax was significantly increased in ISO-induced H9C2 cells in ISO group (*P* < 0.01); and compared with that in ISO group, the expression level of Bcl-2 was significantly increased and that of Bax was significantly decreased in ISO-induced H9C2 cells in mPRDX6 group (*P* < 0.05); compared with that in CON group, the expression level of PPAR-*γ* was significantly decreased in ISO-induced H9C2 cells in ISO group(*P* < 0.01); and compared with that in ISO group, the expression level of PPAR-*γ* in ISO-induced H9C2 cells in PRDX6 and mPRDX6 groups was significantly increased (*P* < 0.01).

## 4. Discussion

Cardiovascular disease is a serious threat to human life, and the heart is easily affected by myocardial pathological factors. The external stimulation to cardiomyocytes will cause the damage or death of them through the way of apoptosis, necrosis, or autophagy to affect the function of the heart. Studies have indicated that heart failure, acute myocardial infarction, and other cardiovascular diseases promote the production of a large number of ROS in the ischemic region, leading to the apoptosis and death of cardiomyocytes, thus aggravating heart diseases [15, 16]. Siwik et al. [17] found that the inhibition on the activity of SOD could increase the concentration of ROS in cardiomyocytes, while the increased ROS could stimulate the hypertrophy of cardiomyocytes, which could be inhibited by ROS scavengers. Therefore, an effective ROS scavenger can alleviate a myocardial injury to some extent. It has been reported that SOD [18] and GPX [19] have a significant therapeutic effect on the myocardial injury. PRDX6, as an important antioxidant protein, can scavenge many kinds of peroxides, such as hydrogen peroxide, fatty hydroperoxides, short-chain hydroperoxides, and phospholipid hydroperoxides to protect the body from oxidative stress damage based on its GPX activity [20, 21]. In contrast to the other PRDX family members and GPX, PRDX6 has a wider substrate specificity, which has attracted our research interest.

It is worth discussing that the other factor of myocardial injury may be that too many ROS can induce the activation of PLA2 [22], and the function of PLA2 is to induce the decomposition of mitochondrial membrane lipids to increase its permeability to accelerate the release of apoptosis factors, inducing the apoptosis of cardiomyocytes themselves to cause the myocardial necrosis [23, 24]. PRDX6 has not only the activity of GPX but also the activity of PLA2. It was considered based on the above reasons that on the one hand, PRDX6 could effectively scavenge the excessive ROS in the body through its GPX activity to protect the myocardium from the oxidative stress damage; on the other hand, PRDX6 might induce the apoptosis and necrosis of cardiomyocytes through its PLA2 activity. Therefore, this study was aimed at further exploring whether the abolition of the PLA2 activity of PRDX6 could be more conducive to the protection of PRDX6 against the myocardial injury. In this study, the recombinant PRDX6 and its mutant mPRDX6 were first obtained, and thena H9C2 cell injury model and an animal myocardial injury model were induced by ISO for studying the effect of both on the myocardial injury.

When myocardial cells are damaged, the ischemia, inflammation, hypertrophy, and necrosis of them will occur [25]. ISO induction is a common method to construct a myocardial injury model. An excessive ISO will increase the oxygen consumption and contraction of myocardium to cause a myocardial injury [26]. In this study, the cell model and animal model of myocardial injury were established by the induction with ISO, and then the repair function of recombinant protein to the myocardial injury was studied. Myocardial cells are the important foundation for repairing myocardial injury, and it is found that recombinant proteins can induce the proliferation of cardiomyocytes, which provides a way of thinking for PRDX6 and its mutants in the treatment of myocardial injury. After the myocardial injury was induced by ISO, the weight of rats was decreased but increased after the treatment with the recombinant protein. H&E staining showed that PRDX6 and mPRDX6 could alleviate the myocardial injury induced by ISO to some extent.

A complete system of antioxidant defense enzymes has been established in organisms during the long-term evolution, including SOD, GPX and CAT [27], of which SOD, the first defense line to oxidative stress injury, can catalyze O^2−^ reaction to generate hydrogen peroxide (H_2_O_2_), and then GPX and CAT can catalyze H_2_O_2_ reduction to H_2_O and O_2_ through different ways [28, 29]. These antioxidases can synergize to protect the body from oxidative damage [30]. Therefore, the expression level of antioxidant enzymes can be used as an important index to evaluate the degree of oxidative damage of cells and tissues. A large number of studies have shown that the expression enzyme activities of SOD, GPX, and CAT in ISO induced myocardial injury model are decreased and increased after the administration of some agents [31–33]. The results of cell and animal experiments in this study were consistent with the above reported results, in which compared with that in ISO group; the expression enzyme activity of SOD was significantly increased, and that of GPX and CAT were improved in mPRDX6 group, suggesting that mPRDX6 could protect the cells from the oxidative stress injury by regulating the expression level of antioxidases, but there was no significant difference in the enzyme activity of antioxidases betweenPRDX6 group and ISO group, which may be related to the PLA2 activity of PRDX6.

Myocardial cells belong to terminal differentiation cells, and the myocardial injury caused by apoptosis is irreversible, so it is very important to prevent apoptosis for the treatment of myocardial injury [34]. The study of Shim et al. showed that the protective effect of ethyl pyruvate on myocardial function was related to its antiapoptosis function [35]. In this study, the apoptosis of cells was induced by ISO, and the number of apoptotic cells decreased after the treatment of PRDX6 and its mutant mPRDX6. We found that the early apoptosis rate of cells was increased and the late apoptosis rate was decreased significantly in PRDX6 group compared with that in ISO group, while both the early apoptosis rate and the late apoptosis rate were significantly decreased in mPRDX6 group. When cells are damaged by oxidative stress, the expression of proapoptotic genes and antiapoptotic genes will be regulated to some extent. Our further research showed that the expression level of Bcl-2 increased and that of Bax decreased significantly in mPRDX6 group compared with that in ISO group, while the expression level of the antiapoptotic protein Bax in PRDX6 group was not significantly different from that in ISO group. PPAR-*γ*, a nuclear hormone receptor, plays an important role in the proliferation, differentiation, apoptosis, and inflammation regulation of cells [36]. Zeng et al. found that MirRNA-128 could inhibit the apoptosis of myocardial cells induced by hypoxia by activating PPAR-*γ* in rats [37]. Han et al. reported that rosmarinic acid could inhibit the apoptosis of myocardial cells induced by ischemia-reperfusion by activating PPAR-*γ* in rats [38]. The results of this study showed that the expression of PPAR-*γ* decreased in H9C2 cells induced by ISO, and the expression of PPAR-*γ* increased significantly after the treatment with PRDX6 and mPRDX6, indicating that mPRDX6 can activate PPAR-*γ* by regulating the expression Bcl-2 and Bax and then inhibit the apoptosis of myocardial cells induced by ISO.

In conclusion, PRDX6 and mPRDX6 recombinant proteins prepared in this experiment showed a certain repair effect in the treatment of ISO-induced myocardial injury in both the cell model and the animal model based on their outstanding antioxidant capacity. However, because the PLA2 activity of PRDX6 may affect the apoptosis to some extent, its function may be inferior to that of mPRDX6. It is suggested that PRDX6 can be modified according to the needs in the future application and development process to play its maximum role. PRDX6 and its mutant mPRDX6 may be a potential drug for the treatment of myocardial injury.

## Figures and Tables

**Table 1:** Weights of rats.

	Initial weight (g)	Terminal weight (g)
CON	182.6 ± 12.9	221.5 ± 13.6
ISO	185.6 ± 14.2	196.8 ± 18.4^∗^
PRDX6	183.9 ± 10.7	208.2 ± 16.1
mPRDX6	180.8 ± 17.3	216.8 ± 14.9^#^

^∗^ *P* < 0.05 vs. CON group and ^#^*P* < 0.05 vs. ISO group.

## Data Availability

The [DATA TYPE] data used to support the findings of this study have been deposited in the [NAME] repository ([DOI or OTHER PERSISTENT IDENTIFIER]). The [DATA TYPE] data used to support the findings of this study are included within the article.
